# The Mannose Receptor in Regulation of Helminth-Mediated Host Immunity

**DOI:** 10.3389/fimmu.2017.01677

**Published:** 2017-11-29

**Authors:** Irma van Die, Richard D. Cummings

**Affiliations:** ^1^Department of Molecular Cell Biology and Immunology, VU University Medical Center, Amsterdam, Netherlands; ^2^Department of Surgery, Beth Israel Deaconess Medical Center, Harvard Medical School, Boston, MA, United States

**Keywords:** C-type lectin, mannose receptor, helminth, schistosoma, trichuris, immune regulation

## Abstract

Infection with parasitic helminths affects humanity and animal welfare. Parasitic helminths have the capacity to modulate host immune responses to promote their survival in infected hosts, often for a long time leading to chronic infections. In contrast to many infectious microbes, however, the helminths are able to induce immune responses that show positive bystander effects such as the protection to several immune disorders, including multiple sclerosis, inflammatory bowel disease, and allergies. They generally promote the generation of a tolerogenic immune microenvironment including the induction of type 2 (Th2) responses and a sub-population of alternatively activated macrophages. It is proposed that this anti-inflammatory response enables helminths to survive in their hosts and protects the host from excessive pathology arising from infection with these large pathogens. In any case, there is an urgent need to enhance understanding of how helminths beneficially modulate inflammatory reactions, to identify the molecules involved and to promote approaches to exploit this knowledge for future therapeutic interventions. Evidence is increasing that C-type lectins play an important role in driving helminth-mediated immune responses. C-type lectins belong to a large family of calcium-dependent receptors with broad glycan specificity. They are abundantly present on immune cells, such as dendritic cells and macrophages, which are essential in shaping host immune responses. Here, we will focus on the role of the C-type lectin macrophage mannose receptor (MR) in helminth–host interactions, which is a critically understudied area in the field of helminth immunobiology. We give an overview of the structural aspects of the MR including its glycan specificity, and the functional implications of the MR in helminth–host interactions focusing on a few selected helminth species.

## Introduction

Parasites have been a great burden to human health throughout many centuries. Parasitic helminths (worms) are a large and important group of parasites that cause diseases, such as ascariasis, filariasis, and schistosomiasis, which are often endemic in tropical areas.

Over the past 20–30 years it has been observed in the Western world that a correlation exists between an effective hygiene and the increase in atopic, autoimmune, and inflammatory diseases. These findings are reflected in the “hygiene hypothesis” (1, 2), and led to the concept that in the developed world the reduction in exposure to helminths affects the immunoregulatory mechanisms of our immune system (3, 4). The existence of a long and close association between helminths and their hosts is proposed to have been the driving force of the co-evolution of helminth and host mechanisms that ameliorate harmful inflammatory responses. These enable helminths to survive and protect the host from excessive pathology arising from infection with these large pathogens (5).

The anti-inflammatory consequences of helminth infections are further supported by the observations that either infection with parasitic helminths or systemic treatment with helminth extracts can reduce the symptoms of allergic diseases (6) and inflammation associated with autoimmune diseases. The latter include inflammatory bowel diseases (7, 8), multiple sclerosis (9–12), or rheumatoid arthritis (13, 14), as well as metabolic disorders such as obesities (15–17), diabetes (18, 19), or atherosclerosis (20). From this perspective, there is some rationale in regarding parasitic helminths, as long as they do not induce obvious pathology, as potentially beneficial commensals rather than dangerous pathogens that need to be expelled. Along this way, infection with helminth parasites is being explored as a potential therapy for a variety of diseases in clinical trials (21).

Increased understanding of the nature of helminth effects on the immune system could enable new treatment options for parasitic diseases, or beneficially modulate inflammatory reactions. Such studies could lead to identification of the molecules involved and promote approaches to exploit this knowledge for future therapeutic interventions. In this regard, there is increasing evidence that carbohydrate-binding proteins, and specifically C-type lectins, play an important role in driving helminth-mediated immune responses (22, 23). C-type lectins are a large family of calcium-dependent receptors and each member has a relatively unique carbohydrate (glycan)-binding specificity. These lectins are abundantly present on immune cells that shape host immune responses and collectively they can recognize a wide variety of glycans.

## Helminth Infection and Helminth-Induced Immune Reactions

Infection with parasitic helminths typically induces a type 2 (Th2) immune response and promotes the generation of alternatively activated macrophages (AAMs) and eosinophils. Soon after infection, innate responses are initiated by many different cell types [including antigen-presenting cells such as dendritic cells (DCs) and macrophages], which, upon encountering the invading parasites, promote the suppression of T-cell-driven protective immune responses and a shift to Th2 responses. The helminth-driven Th1/Th2 immune responses are controlled through the generation of regulatory networks, which can include FoxP3+ regulatory T cells (Treg), anergic/hyporesponsive T cells, regulatory monocytes/macrophages, and/or B cells.

It is possible that evolution of different types of helminths has resulted in relatively similar pattern of immune responses in infected hosts. Many different molecules, receptors, and host cells cooperate and interact, generating mechanisms that have evolved to achieve a balance between host and parasite, dependent on the living environment and biology of the parasites. To dissect the different molecular mechanisms and signaling pathways involved, experimental data with isolated soluble products are essential. Several of such helminth products have been purified and studied in animal models and *in vitro* assays with antigen-presenting cells (24–29). These parasite-derived molecules include secreted glycoconjugates, e.g., glycoproteins and glycolipids, which play important roles in host immune modulation. The helminth-derived glycans can interact with immune cell-expressed C-type lectins [termed C-type lectin receptors (CLRs)] and other glycan-binding proteins, such as galectins, and these interactions help to shape the innate and adaptive immune responses (22, 23, 30). Because helminths do not express sialic acid, they do not appear to interact with the Siglec family of sialic acid-binding lectins on immune cells. Dendritic cells express many different CLRs, including DC-SIGN, Dectin-1, MGL, and the mannose receptor (MR); their expression can vary within distinct DC subsets. CLRs can act as endocytic and/or signaling receptors, and play major roles in both innate and adaptive immune responses often in concerted action with other CLRs and/or toll-like receptors (TLRs) (31–33). One of the best studied CLRs is the human DC-SIGN (31, 34), which typically binds glycans containing terminal fucose or mannose residues (35), such as fucosylated glycans of *Schistosoma mansoni*, including Lewis-X [Galβ1-4(Fucα1-3)GlcNAc-], LDNF [GalNAcβ1-4(Fucα1-3)GlcNAc-], and the schistosome-specific pseudo-LeY ligand [Fucα1-3Galβ1-4(Fucα1-3)GlcNAc-] (35–38). Remarkably, DC-SIGN induces distinct signaling pathways dependent on the type of glycan that is recognized (39, 40). Similar to DC-SIGN, the MR typically recognizes mannose- and fucose-containing glycans in both trematode and nematode parasites, but its glycan specificity and functions are less well understood.

## The Mannose Receptor

### Structural Properties of the MR

**Figure 1:**
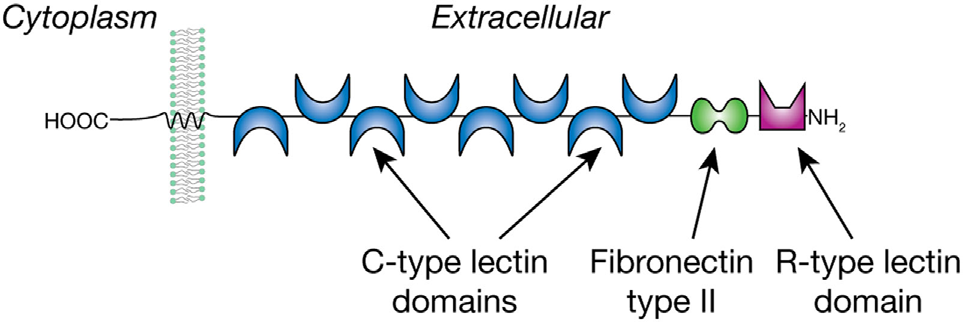
The mannose receptor is a type 1 membrane glycoprotein with multiple C-type lectin domains that can bind mannose- and fucose-containing glycans and an R-type lectin domain that binds sulfated Gal/GalNAc residues.

The MR is expressed by a selected population of myeloid cells and non-vascular endothelium and has been implicated in helminth-induced modulation of host immune responses. The MR is a type I membrane glycoprotein of 165 kDa that is comprised of a cytoplasmic domain of 45 amino acids and three types of extracellular domains as shown in Figure 1. These domains are an N-terminal cysteine-rich domain, followed by a fibronectin type II repeat (FNII), and eight consecutive C-type lectin-like domains (CRDs) (41). The MR was originally described as an endocytic receptor with a broad binding specificity for both microbial and endogenous ligands and constantly cycles from the cell surface to the cytoplasm (42, 43). More recently, there is evidence that the MR is also involved in cellular activation and signaling. However, the signaling activity of the MR is unusual, since the receptor does not have clear signaling motifs in its cytoplasmic domain; thus, the mechanisms and potential signaling pathways may involve the action of co-receptor(s) and are poorly understood (44).

### Glycan Specificity of the MR

The MR is unique among the CLRs in that it consists of multiple carbohydrate-recognition domains (CRDs) (Figure 1). The N-terminal domain is an R-type domain that binds in a calcium-independent manner to glycans that have a non-reducing terminal 3-*O*-sulfated galactose or 3/4-*O*-sulfated-*N*-acetylgalactosamine (45). The FN II domain is involved in binding collagens (46). The MR was eponymously named by its property of binding to mannose, which is mediated within the C-type lectin domains 4–8. Fibroblast expression studies showed that CRD 4 has the major affinity for carbohydrate, whereas CRDs 5 and 7 appear to contribute to the binding capacity of mannose-containing glycans. Removal of CRDs 1–3 did not affect affinity for the ligands tested (47), and their roles, if any, in glycan binding is unknown. *In vitro* binding studies with the MR showed its preferential but weak interaction with both the monosaccharides Man and Fuc above other monosaccharides (48). With more complex glycans the MR shows a preference for Manα1-6Man-R and Manα1-3Man-R compared to α1-2/4-linked Man residues, whereas the branched mannotrioside Manα1-6(Manα1-3)Man-R showed the highest affinity to the MR. Of the fucosylated ligands tested, Fucα1-6GlcNAc-R showed a similar affinity as the branched mannotrioside, whereas binding to Fucα1-2Gal-R was lower and α1-3/4-linked Fuc was not tested. The latter linkage is found in the Lewis antigens to which the MR does not bind (49), in contrast to the C-type lectin DC-SIGN (37), which is also expressed by DCs and shares with the MR a preference for mannose/fucose/GlcNAc. The MR selectivity for Manα1-3/6Man corresponds well to its function as a pathogen receptor, considering the abundance of these termini in yeast mannans, and the presence in helminths of paucimannose-*N*-glycans such as Man_3_GlcNAc_2_-Asn (23). It is likely that the multivalent nature of the MR facilitates high avidity interactions with multivalent or repetitive glycan-ligands, which occur in many microorganisms, fungi, and parasites (48). Whereas multivalent binding by most CLRs is mediated by multimer-forming of lectin molecules, the presence of multiple CRDs in the MR is thought to promote its multivalent binding within a single MR molecule. This implies that the binding affinity of the MR highly depends both on valency and structural characteristics of a particular glycoconjugate (47).

The glycosylation of the MR may further fine-tune its binding to ligands (50). The MR contains many Asn-linked N-glycans, and their structures in the mouse appear to be tissue specific (51). Terminal sialylation of glycans on the MR is of special interest, since it has been suggested that this may affect the MR binding properties to mannosylated glycans, whereas non-sialylated or neutral glycans might affect the avidity for sulfated carbohydrate ligands (50). Such differential glycosylation of the MR might not only influence its binding properties to exogenous ligands but might also influence its interactions with other receptors on the cell membrane, thereby possibly modulating MR functions.

### Expression of the MR on Immune Cells

The MR is primarily expressed on human and mouse DCs and macrophages (MF), but it is also found on other cells, such as non-vascular endothelial cells (44). Interestingly, the MR is largely found intracellularly in membranous structures, and only 10–30% is expressed at the cell surface under steady state conditions (43); this is consistent with the recycling and internalization nature of the receptor. The Th2 cytokines interleukin (IL)-4, IL-13, and IL-10 (52–54) as well as prostaglandins PGE1 and PGE2 (55) upregulate MR expression on murine macrophages; in human macrophages generated *in vitro* culture with human serum, activation by treatment with IL-4 results in significantly increased MR expression (56). The MR is expressed at low levels on naïve monocytes. Monocytes constitute around 10% of total leukocytes in blood and are key players of the human innate immune response. Blood-derived monocytes are an independent cell lineage that has the ability to differentiate into specific DC and macrophage populations, which often constitutively express the MR. In monocytes, the expression of the MR is induced upon maturation (44), and specific (pro-inflammatory) subsets of monocytes have been reported to be MR positive (57). Interestingly, a sub-population of monocytes with an enhanced expression of the MR has been identified in patients with asymptomatic filarial infection; such expression is correlated with enhanced expression of the suppressor of cytokine signaling-1 (SOCS-1) and the cytokines IL-10 and transforming growth factor β (TGFβ) (58). We recently observed a similar monocyte phenotype in helminth-infected Ethiopian individuals (unpublished observation). Furthermore, human monocytes treated *in vitro* with soluble components (SPs) of the whipworm *Trichuris suis* induce a sub-population of anti-inflammatory patrolling monocytes with enhanced CD16, IL-10, and MR protein expression (59). These data indicate that interaction with helminth components, either directly or indirectly *via* the induction of Th2 cytokines, can induce expression of the MR on monocytes. Such modulated monocytes may differentiate to AAMs, as are known to be induced by helminths (60). Indeed, human monocytes treated *in vitro* with *T. suis* SPs differentiate into a subset of macrophages with enhanced AAM properties, including elevated MR expression and IL-10 production (61). An interesting possibility is that the helminth-induced MR expression on AAMs may be relevant for the known role of AAMs in wound healing. A common property of helminths is that they need to migrate in the hosts as part of their life cycles, and this causes extensive tissue damage. The ability of helminths to thus limit host-damage may promote their survival in the hosts. In summary, these data indicate that helminths modulate the phenotype of human blood monocytes, which in turn may lead to the generation of AAMs expressing the MR.

## The MR in Helminth-Mediated Immune Responses

Whereas enhanced expression of the MR is observed upon contact with helminths as described above, the role of the MR in modulating immune responses is still unclear. The MR interacts with and internalizes components of several helminth species, and this is often associated with the induction of anti-inflammatory or Th2 responses (Table 1).

**Table 1:** Interaction of the mannose receptor (MR) with helminth components, and immunological parameters.

Helminth	Components	Effects	Reference
*Schistosoma mansoni*	SEA	Interaction with MR (h, m), internalization in dendritic cells (DCs) (h)	(62, 63)
	Omega-1	Internalization of Omega-1 by MR in DCs (h)	(64)
	SEA	MR-dependent expression of suppressor of cytokine signaling-1 and SHP1 on DCs (h)	(65)
	Larval E/S	Binding Larval E/S products by MR; reduction INF production, stimulation Th2 responses (m)	(66)

*Fasciola hepatica*	E/S products	Induction high levels of transforming growth factor β and interleukin (IL)-10 in macrophages (m)	(67)
	Tegumental coat proteins	Binding to BMDCs (m)	(68)

*Trichuris suis*	SWP	Inducing enhanced motility of monocytes (h)	(59)
	SWP	Enhancing IL-10 and reducing CCR2 expression on monocytes (h)	(59)

*Trichuris muris*		Binding of BMDM and production of IL-6 (m)	(69)

*Trichinella spiralis*	SP L1 larvae	Binding to macrophages, stimulation of NO secretion (m)	(70)

*Ascaris suum*	High MW SWP	Inhibition LPS-induced BMDCs maturation and *in vitro* T cell proliferation (m)	(71)

*h, human; m, mouse; SEA, soluble egg proteins; SWP, soluble worm products; E/S, excretory secretory products; MW, molecular weight*.

### Flatworm Trematodes Interacting with the MR

The interaction of the MR has been reported both with the bloodfluke *S. mansoni* and with the liver fluke *Fasciola hepatica* (Table 1). *S. mansoni* is a human parasite causing schistosomiasis (bilharzia), affecting millions of individuals especially in tropical areas (72). *S. mansoni* can also infect rodents, which are often used as model system to study the immunobiology of the disease. *F. hepatica* primarily infects sheep and cattle causing fascioliasis, but is also an important emerging pathogen of humans (73).

*Schistosoma mansoni*—Th2 polarization by infection with *S. mansoni* and exposure to *S. mansoni* antigens involves the induction of tolerogenic DCs and the expansion of regulatory cell populations (including IL-10 secreting and Foxp3-expressing Tregs) (74–76). The immune response against *S. mansoni* infection begins at the earliest stage of infection, when cercaria gain entry to the mammalian host *via* the skin, which initially stimulates the innate immune response. During transformation from cercariae to schistosomula within 72 h after infection, the parasite secretes large amounts of highly glycosylated components, termed excretory/secretory (E/S) products. Mononuclear phagocytic cells in the skin internalize E/S products released by the schistosomula *via* the MR (66). In addition, it was shown that the ligation of the MR by *S. mansoni* larval E/S products has a major role in limiting the production of pro-inflammatory cytokines (66), which may prime the immune system for the subsequent development of a Th2 response.

After maturation of the larvae to adult worm pairs of female/male, eggs are deposited that secrete soluble egg components. One of these components is Omega-1, a major secreted egg glycoprotein RNase, which is capable of inducing a Th2 response (25, 77, 78). It has been proposed that the MR on DCs is essential for internalization of Omega-1, which subsequently acts as an RNase to degrade RNA thereby impairing protein synthesis (25, 64). Intraperitoneal injection of obese mice with Omega-1 resulted in a Th2 immune response in the white adipose tissue, improving glucose tolerance and induction of a transient delay in weight gain (79). Whereas IL-33 release from cells in the adipose tissue was mediated by the RNase activity of Omega-1, its ability to improve metabolic status was shown to be dependent upon effective binding to the MR (79).

We recently showed that SEA, both untreated and heat-treated (in which RNases and thus Omega-1 activity were eliminated), potently suppressed LPS-induced TNF and IL12 production and upregulated SOCS-1, SHP-1, and OX40L expression in human DCs (65); these are phenotypic and functional changes in DCs associated with Th2 polarization. Remarkably, treatment of SEA with periodate (PI) (in which glycans are oxidized and lose their recognition potential), causes a loss of the inducing activity, suggesting an important role of SEA glycans in regulating DC function. Similarly, CD4+ T cell proliferation was suppressed by the addition of DCs primed with either untreated or heat-treated SEA, but suppression was not observed by using PI-treated SEA (65). The SEA-induced upregulation of expression of SOCS-1 and SHP-1 appeared to be MR-dependent. These data indicate that RNase activities within SEA are not essential to induce Th2 polarizing DCs in the human system; however, it is possible that glycans linked to Omega-1 and/or other MR-ligands trigger the MR to induce inhibition of pro-inflammatory responses, perhaps similar to the larval E/S products (66).

Many reports have described a potential role of parasite-derived glycans in modulation of schistosome-mediated immune responses (39, 66, 80–82). The observation that PI-treated SEA has a strongly decreased ability to modulate DC function, compared to heat-treated and untreated SEA, also indicates that glycans within SEA play an important role in polarization of DC-mediated immune responses (65). The observation that Omega-1, in contrast to SEA, has no potential to inhibit T-cell proliferation (83) suggests that SEA contains additional components that contribute to modulation of the host’s immune response; thus, it will be important to identify the SEA components that are responsible for these properties. One possibility is that the lipid mediator prostaglandinE_2_ (PGE_2_) contributes to SEA-induced immune responses. SEA preparations have been shown to contain the lipid mediator PGE_2_ (27, 84), and PGE_2_ has been shown to have the potential to induce Th2 responses (27, 85). Remarkably, it appears that the activity of PGE_2_ is PI-sensitive (27), which shows that deducing a role for glycans based only on PI sensitivity of the putative compounds should be regarded with caution. In conclusion, there may be several pathways and multiple schistosome components mechanistically involved in the suppression of inflammatory responses and Th2 polarization, some of which essentially involve a role of the MR.

*Fasciola hepatica*—As observed with many other helminths, infection with *F. hepatica* leads to downregulation of Th1 immune responses and the generation of Th2 immune responses in mice (29, 86). During infection, the parasites release a myriad of different products (E/S products and tegumental antigens) that downregulate Th1 responses and promote Th2 responses, including development of AAMs with immunomodulatory potential (29, 87). Macrophages stimulated with *F. hepatica* E/S products show enhanced MR, Arg-1, TGF-β, IL-10, and PD-L1 expression and a reduced potential to respond to LPS activation (67, 88). Furthermore, blocking the MR with the mannan hapten or an anti-MR blocking antibody resulted in a partial loss of the macrophages’ inflammatory phenotype. Interestingly, similar effects were observed when mice were intraperitoneally injected with mannan before being infected (67).

*Fasciola hepatica* tegumental antigens (FhTeg) enhance expression of the negative regulator SOCS3 (89) and the MR (90) on BMDCs, which may contribute to its immune modulatory properties, such as the induction of T-cell anergy or T-cell hyporesponsiveness (90). Interaction of FhTeg, which contains glycoproteins with oligo-mannose-type glycans, with BMDCs was partly MR-dependent (68). On the other hand, the ability of FhTeg to induce SOCS3 or suppress cytokine secretion from LPS activated BMDCs appeared not to be MR-dependent, as was demonstrated by the use of MR-deficient BMDCs (68), indicating that other mechanistic pathways are involved. The enhanced MR expression on the FhTeg-treated BMDCs has been suggested to be involved in induction of T-cell anergy. DC-CD4+ T-cell communication appeared to be MR-dependent, as was deduced from a reduced ability of MR-deficient BMDCs to enhance expression of the anergic markers GRAIL and CTLA4 on CD4+ T-cells, and a reversal of the suppression of IL-2 and IFN-γ compared to mock-treated BMDCs (90). These data illustrate a role for the MR in the immunoregulatory properties of both murine macrophages and BMDCs upon interaction with *F. hepatica* components.

### Whipworms Interacting with the MR

Parasitic nematodes of the order Trichocephalida (whipworms) contain several genera of medical importance including *Trichuris* and *Trichinella* species. Human infection with *Trichuris trichuria* and *Trichinella spiralis* typically occurs after ingestion of contaminated food. *Trichuris muris* is often used as a natural mouse model of *T. trichiura*. The pig whipworm *T. suis* has strong anti-inflammatory properties (27, 91, 92) and transient infection with these parasites, which are not able to reproduce and lack long-term survival in non-pig mammals, are being investigated as a natural treatment for human inflammatory diseases, such as inflammatory bowel disease and multiple sclerosis (21). Studies with *T. muris* in different mouse models and *T. suis* infection in pigs have shown that a Th2-dominated immune response is required for worm expulsion (93, 94), whereas the development of a Th1 response leads to host susceptibility (94).

A Th2-dominated response includes the generation of AAMs which typically express the MR. *T. muris* E/S products contain components that bind to the MR; however, a functional role *in vivo* for the MR in worm expulsion could not be demonstrated (69). Knockdown of the MR revealed a role of the MR in the production of IL-6 by the AAMs, but no effect on the expulsion of the parasite (69). This suggests that either the MR may not be involved in expulsion of the parasite or alternative pathways compensate for the loss of the MR. Interaction of *T. spiralis* L1 larvae with the MR expressed on the surface of peritoneal macrophages did not mediate IL-6 secretion, but resulted in an enhanced NO production, suggesting that the MR contributes to macrophage activation.

Human DCs bind soluble components of *T. suis via* C-type lectins including the MR (59, 91). To date, no clear role for the MR has been demonstrated upon interaction of *T. suis* components with DCs (unpublished observations). However, monocytes showed an enhanced expression of the MR upon treatment with *T. suis* components associated with the generation of a non-classical phenotype (59). In addition, treatment of endothelial cells with *T. suis* resulted in an enhanced motility and reduced trans-endothelial migration in an *in vitro* model of the blood–brain barrier. The presence of MR blocking antibody significantly inhibited the *T. suis*-induced patrolling behavior of monocytes and rescued the *T. suis*-induced reduction in monocyte trans-endothelial migration. In addition, the MR can induce these properties in monocytes *via* downstream signaling including the action of protein kinase C (PKC) (59). This indicates that the MR is critically involved in the monocyte modulation.

## Discussion and Future Prospects

The MR is an important CLR that interacts with a number of products generated by a variety of helminths, and clearly plays a role in modulating host immune responses, but many questions remain about its functional mechanisms. Due to its presence on different cells in the immune system, ligation of the MR might lead to different signaling consequences, but whether the MR can signal alone or requires co-receptors is unknown. In addition, the presence of multiple carbohydrate-binding domains in the MR allows differential binding of natural glycan ligands and differential effects. Little is known about the MR binding specificity to natural ligands of pathogens including helminths, and this is an important aspect to address. The ambiguity of the MR role is also illustrated by the observation that DCs, primed with some natural ligands of the MR, such as MUC III, biglycan, and *Mycobacterium tuberculosis* mannosylated lipoarabinomannan, inhibit the generation of Th1-polarized immune responses, whereas other ligands that also bind the MR, such as thyroglobulin, had no effect (95).

The observation that glycosylation of the MR itself influences its glycan-binding properties, suggests that the function of the MR can vary dependent on the cells that express the lectin and their activation status and ability to glycosylate the MR. It is known that in DCs, for example, their glycosylation dramatically changes during cellular activation (96), which may result in changes of the glycosylation state of the expressed MR, but this has not yet been demonstrated.

Since the cytoplasmic domain of the MR has no clear signaling motifs, it has been assumed that the MR cannot directly induce downstream signaling upon ligand binding. We recently demonstrated, however, that the MR is critically involved in PKC signaling in monocytes (59), and many of the effects observed for MR ligation imply its signaling potential. The most likely explanation is that the MR may be needed for concerted action with another receptor that may be more directly involved in signaling, and that the primary role of the MR may be in capturing and/or internalizing a ligand. For example, collaboration of the MR with Dectin-1 has been suggested to be important in inducing high levels of TGF-β and IL-10 in macrophages upon stimulation with *F. hepatica* E/S products (67). Furthermore, the MR and TLR2 are both critically involved in pro-inflammatory cytokine production by human monocytes in response to *Pseudomonas aeruginosa* infection (97). Thus, the MR may indirectly influence signaling cascades in immune cells, but the exact mechanism of how this collaboration takes place is unknown.

The MR is one of the most unique CLRs produced by animals. The ability of this receptor to bind a wide variety of mannose- and fucose-containing ligands puts it at the forefront of the innate immune response to pathogens rich in such glycan signatures. While there are many aspects of MR functioning and glycan recognition yet to be discovered, there are exciting translational opportunities as the glycan ligands that regulate MR activity are identified and allow us to exploit its anti-inflammatory and regulatory functions.

## Author Contributions

All authors listed have made a substantial, direct, and intellectual contribution to the work and approved it for publication.

## Conflict of Interest Statement

The authors declare that the research was conducted in the absence of any commercial or financial relationships that could be construed as a potential conflict of interest.
